# The (In)effectiveness of Attention Guidance Methods for Enhancing Brand Memory in 360° Video

**DOI:** 10.3390/s22228809

**Published:** 2022-11-15

**Authors:** Jani Pavlič, Tina Tomažič

**Affiliations:** Faculty of Electrical Engineering and Computer Science, University of Maribor, 2000 Maribor, Slovenia

**Keywords:** product placement, visual attention, attention guidance methods, diegetic guiding cues, brand recall, brand recognition

## Abstract

Sensing and remembering features in visual scenes are conditioned by visual attention and methods to guide it. This should be relevant in terms of product placement, which has become an important part of incorporating brands into different mass media formats with a commercial purpose. The approach can be challenging in 360° video, where an omnidirectional view enables consumers to choose different viewing perspectives, which may result in overlooking the brands. Accordingly, attention guidance methods should be applied. This study is the first to explore diegetic guidance methods as the only appropriate guiding method for an unobtrusive and unconscious nature of product placement. To test the effectiveness of three different diegetic guiding methods, a between-subject design was employed, where the participants were assigned randomly to one of four videos with the same scene but different guiding methods. The findings show and explain the discrepancy with studies on guiding attention in other contexts, as there were no significant differences between the guiding cues according to brand recall and brand recognition. The results also indicate a significant influence of brand familiarity on brand recall in 360° video. The article concludes by providing limitations, future research directions, and recommendations for audiovisual policy.

## 1. Introduction

Visual attention refers to sensing and interpreting relevant information and ignoring the rest by focusing on a certain aspect of the visual scene. It has had an increasing research interest across various fields throughout history [1], including the studies of Human-Computer Interaction (HCI) [2] and Marketing [3]. In this vein, 360° video has become popular recently, due to its immersive viewing experience and omnidirectional view [4]. Its consumption on Youtube increased during the COVID-19 pandemic [5]. The massive support of Information Technology systems has caused substantial interest in exploring the medium from different aspects [6,7], among others, from the perspectives of guiding visual attention [8,9,10,11] and product placement [12,13,14].

As interrupted cognitive processes in advertising are not tolerated well by users [15], product placement has become a crucial part of marketing strategies to influence brand recall and merge narratives with the brand without disturbing the story [16]. It is a relatively old approach, as the first brands were placed in the 1920s [17], and the placements have grown in 2021 across various formats, such as television, film, digital media, video games, print media, and music [18]. The literature on product placement started occurring in the 20th century, with a focus on the paid entry of a branded product in movies or television programs [17], and achieved a peak number of publications in 2004, continuing with number fluctuations until 2011. In recent years, product placement in video games, or “in-game” advertising, has been examined frequently [19], and product placement in more advanced and immersive media technologies has also started taking place [14], such as product placement in Virtual Reality (VR) games [20], 3D and 4D movies [21], and 360° video [12].

360° video represents a promising, yet challenging format for placing brands [13], as consumers are free to choose different viewing perspectives. Visual attention should, thus, be guided to enhance the possibility of sensing and memorizing the brand. On one hand, visual attention has been explored in product placement studies for over-the-top media services [22] and video games [23]. On the other, studies explored the issue of directing attention in 360° videos on a more general level [9,10,24,25,26]. However, no research to date has examined the effectiveness of guidance methods in terms of brand recall and brand recognition in 360° video, which should be considered separately, due to the unconscious nature of product placement [27].

This study focuses on the effectiveness of subtle diegetic guidance methods for enhancing placed brand memory in 360° video by examining the research question: Which diegetic guidance methods are the most effective for product placement in 360° video? The reasons for the appropriateness of exploring diegetic guiding in this context are threefold: (1) Diegetic guiding cues derive from the film`s theory, and are considered as a part of the scene [9]; (2) Their processing is in line with the unconscious processing of product placement [27], and (3) They are less likely to disrupt the natural process of watching and experiencing the story [28] and provide better user experience in cinematic virtual reality [8].

This is the first study exploring the influence of diegetic guidance methods on product placement effectiveness in 360° video, and it is the first that addresses the issue of omnidirectional 360° view in terms of (over)looking the desired viewing perspective and placed brands. It is essential to explore the issue from the perspective of visual attention and guidance, as they can influence viewers’ memory and choices [29,30]. In addition, the strength and novelty of the article are reflected through examination of the not yet established, but growing, format of 360° video [31]. The latter has become important in the context of advertising [32,33], but scarcely explored in product placement, which is less irritating than traditional advertising and still very popular in various media formats [18]. Accordingly, the article has the following contributions: (1) It exposes and defines the issue of visual attention and diegetic guiding methods in terms of 360° video and brand memory in product placement, (2) It provides a comparison of the effectiveness of diegetic guiding methods for placed brands` recall and brand recognition, (3) It confirms the statistically significant influence of brand familiarity on brand recall in this context, and (4) It provides concrete future research directions and policy implications.

This content is structured as follows. Section 2 describes the fundamental theoretical concepts of Visual Attention, 360° video, and Product Placement, and provides related studies in these areas, along with an identified research gap and stated hypothesis. Section 3 summarizes the employed methodology, including the sample, procedure, and measuring instrument. Section 4 comprises the results, and descriptive and inferential statistics to test the hypothesis. A discussion, interpretation of the given results, future research directions, limitations, and policy implications are provided in Section 5.

## 2. Theoretical Background

### 2.1. Visual Attention in 360° Video

Sensations in the human eye are conditioned by a retina, comprised of a central fovea and a periphery [34]. Sensing the information with the human eye, however, is not a sufficient condition for visual attention. The latter turns looking into seeing based on prior knowledge and relevant information. It is, in general, divided into (1) Spatial attention which refers to focusing on a particular location; (2) Featured-Based Attention (FBA) for recognizing colors, orientation, and motion direction; (3) Object-based attention with the guidance of an object structure [1]. The fundamentals of visual attention guides can be explained through the feature-integration theory [35], where features are detected and gathered early (e.g., color, orientation, brightness), and are later combined and perceived as an object.

Capturing and attracting viewers’ attention can generally be classified as bottom-up and top-down factors. The former refers to objects’ visual properties (e.g., color, shape, orientation), while the latter represents a task or a goal that differs between individuals [30]. Visual attention is limited by two types of processing: The processing speed of visual information and visual working memory for remembering the objects [36,37], where the latter are stored as integrated objects of color and orientation [38]. The eye-tracking methodology is suitable for the studies of exploring visual focus, as there is a relationship between the human gaze and the focus of attention. However, the data about eye movements do not always reflect visual attention and visual working memory [34,39], which calls for more suitable measurement instruments, such as variables of brand recall and recognition in terms of product placement [27].

Guiding viewers’ attention in 360° video is similar to the real world. An omnidirectional view provides three degrees of freedom and an immersive viewing experience [4,40], but the same factors may cause difficulties in guiding their attention in the desired direction, where attention guidance methods or cues can be applied to solve the issue [25]. Diegetic and non-diegetic cues’ categorization is relevant for our study, deriving from the film theory [41]. Diegetic guiding methods or cues are considered part of the scene, such as the motion of the main character or sound from the scene, whereas non-diegetic guiding cues are not part of the captured scene, such as graphics, background music, or voice-over. Diegetic cues are also world-referenced or connected to the virtual world, while non-diegetic cues are, typically, screen-referenced (moving with the screen), but they can also be world-referenced, such as virtual objects in augmented reality, or animated graphics in interaction with the environment [9].

Diegetic elements can be seen and heard by both viewer and the character and are part of the story. To capture and direct viewers’ attention, audiovisual and film producers can use different diegetic methods incorporated into the story world, such as moving protagonists and lights [8,25]. Visual attention is not always directed by visual guiding methods but can also be stimulated by sound. Even if the sound is not spatial, viewers are directed by searching for its source [9]. The diegetic cues typically result in subtleness, where users are guided, but unaware of the guiding method. From this perspective, methods are categorized as subtle and overt. Subtle resemblances of diegetic methods work on an unconscious level, while overt is non-diegetic and noticed more easily by the user. We recognize subtle techniques as more appropriate for capturing attention in 360° video and VR systems, since they are less likely to distract the natural process of watching or interacting with the environment than overt methods [28]. In some cases, it is hard to distinguish between subtle and overt methods, especially in the effects of some post-production techniques and corrections (e.g., saturation, exposure, hue), where the categorization depends on the degree of modification [25].

### 2.2. Product Placement

Product placement is an important form of advertising where directing visual attention can play a crucial role in detecting and recognizing placed brands. It differs from traditional advertising since commercial content and program content are not separated strictly [42]. It can be defined as “the paid inclusion of branded products or brand identifiers, through audio and/or visual means, within mass media programming”. [43]. Throughout history, it was typical to influence movie or TV audiences via planned and unobtrusive placement [17], but the practice has started occurring in other forms, such as novels, music videos, games [19], and even more advanced media formats of virtual reality, 360° video and other immersive media [14]. The Audiovisual Directive in Europe tolerates PP in all audiovisual media services except for news, current affairs programs, religious programs, and children’s programs [44]. In this light, disclosures, or product placement logos at certain parts of the program are important for protecting consumers from underground advertising and informing the audience about the commercial message [45] by raising the persuasion knowledge [46]. Disclosure types are different across states, however, content analyses of prior studies show that the leading TV broadcasters in the EU do not pay much attention to the disclosure [47], and there is a scarcity of research exploring it in emerging media technologies (e.g., 360° video).

An integrative conceptual model has been developed for various factors influencing product placement outcomes [27]. The latter comprises execution factors, individual-difference factors, and processing depth/context setting, influencing product placement effectiveness. Several variables from the model are relevant to the present study. In terms of execution factors, the opportunity to process the placement refers to brand prominence and the duration of its exposure [48], while placement modality provides information about visual, auditorial, or plot placement [49]. Prior familiarity with the brand is an important individual difference factor, since more familiar brands can result in higher recall rates [50,51]. Processing depth relates to the level of consciousness of processing the brand, and typically, brands in product placement are processed with a lower level of consciousness than those in conventional advertising [27]. The effects of placement are categorized into cognitive, affective, and conative outcomes, where the cognitive category represents brand memory, with brand recall and brand recognition as the most frequently used measures [14]. They are also the most suitable for the current study, as brand memory indicates the effectiveness of directing viewers’ attention to the brand. Affective responses are more related to consumers’ attitudes toward the brand, while conative responses represent consumers’ behavior, such as brand choice or purchase intentions [27]. Those are more relevant for different marketing goals, where the brand has already been detected.

In addition to the mentioned factors, technology-related variables can influence product placement effectiveness, where the impacts differ according to the technology condition [14], which is also relevant for 360° video, that can be played on various devices. It is a challenging format for product placement, due to its omnidirectional view, where viewers can choose different perspectives, resulting in a higher possibility of overlooking the placed brand. Our study thus tests the effectiveness of diegetic guiding methods for directing viewers’ attention.

### 2.3. Related Work and Hypothesis Development

Prior studies explored the effectiveness of several methods for capturing and directing viewers’ attention in 360° video. Researchers [8,25,52] compared the effectiveness of diegetic and non-diegetic methods in 360° video or cinematic virtual reality (CVR), and marked diegetic cues as more helpful, while non-diegetic cues were substantiated to lower the sense of presence, which is a crucial concept of virtual reality and immersive experience [53]. In terms of user experience, diegetic cues (moving objects, moving protagonists, and small gestures such as head pointing) turned out to have better ratings than non-diegetic cues (forced rotation and object or environment manipulation) [8].

Another study substantiated the positive influence of sound (spatial and non-spatial), moving objects, and lights on guiding viewers’ attention, where a beginning of a new scene turned out to be the most challenging [9]. Similarly, the study on viewers’ attention in the 360° video by means of HMD [26] shows that the combination of visual and audio cues is more potent than only visual, which was true even without fully spatialized audio. Regarding visual cues, moving characters turned out to be the most unobtrusive for directing attention toward the target. In addition, there was a positive response from participants being addressed by characters in the scene.

Despite prior studies exploring the cues for guiding viewers’ attention in 360° and virtual environments [9,10,24,25,26], no previous research has investigated the differences in guiding cues for effective product placement approaches in terms of brand memory. Only two studies to date have explored product placement in 360° video [12,54], considering other influences on brand perception (e.g., dialogic engagement) without focusing on guiding methods for directing viewers’ attention. Related studies on visual attention and product placement were explored in the over-the-top media service [22] and video games [23] by using the eye-tracking methodology, where no guiding cues were explored.

From the marketing perspective, there is a need for research in this context, due to the omnidirectional nature of 360° video [55], where placed brands can be overlooked easily. The issue must be explored separately from related studies on guiding cues, as placed brands are typically processed unconsciously and are of secondary importance to the audience [27]. Thus, capturing and directing attention through guiding methods may not necessarily influence the brand memory, but it can guide viewers’ attention in the right direction and increase possibilities for cognitive focus. The present study fills the research gap by comparing the effectiveness of three different diegetic guiding cues in 360° according to the brand memory. Accordingly, the research question was formed: Which diegetic guidance methods are most effective for product placement in 360° video? In this vein, we developed a hypothesis that defines the tested relation between diegetic guidance methods and brand memory.

**H1.**  *Respondents in the groups of diegetic guidance methods will have significantly higher (a) brand recall and (b) brand recognition scores than those in the control group.*

## 3. Method

### 3.1. Research Design

**Figure 1:**
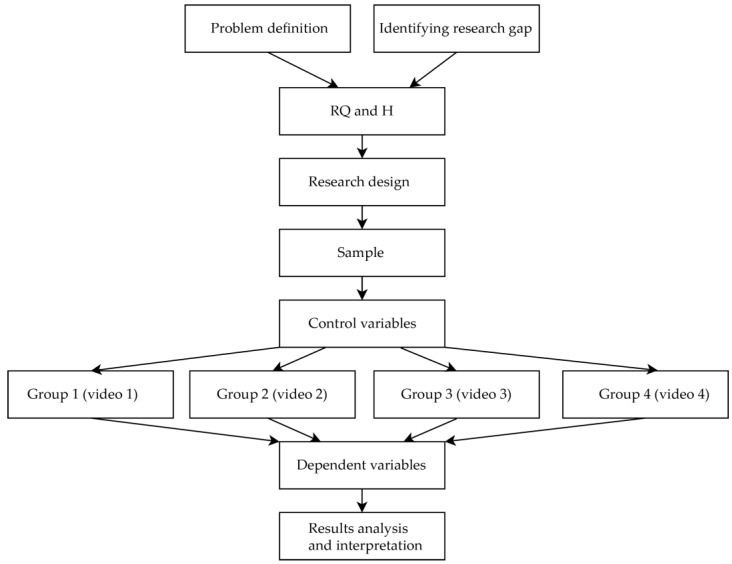
Research flow diagram.

The research design consists of different stages, depicted in Figure 1. Previous sections consider the research focus with problem definition and research gap identification, research question, and hypothesis. This section describes important elements of the methodology for obtaining the data.

We used non-probability convenience sampling on users of the SurveyCircle platform, who were invited to watch a 360° video and fill in the questionnaire. Firstly, all the respondents answered the questions regarding control variables (see Section 3.3). Secondly, a between-subject design was applied, where each participant was assigned randomly to one of four videos with guidance methods. Lastly, respondents from all groups answered the same questions regarding the dependent variables of brand memory.

**Figure 2:**
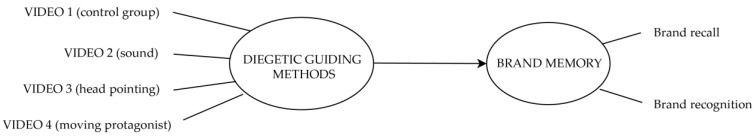
Proposed Research Model.

We proposed a model of differences between diegetic guidance methods according to brand memory, namely, brand recall and brand recognition (Figure 2). In this vein, diegetic guidance methods/cues are independent variables, whereas brand memory is a dependent variable, consisting of two empirical indicators—brand recall and brand recognition.

### 3.2. Videos and Guidance Methods

To compare the effectiveness of different diegetic guidance methods, we have produced four 360° videos 90 s in length. The videos were produced with a monoscopic 360° camera in a controlled environment of a television studio. We did not apply any special editing methods, as they might have reduced the viewing experience [56]. All the videos depicted the same scene, but different diegetic methods for guiding viewers’ attention toward the brand on the protagonist’s t-shirt. We provided short synopses, along with guidance methods and screenshots of chosen perspectives from the 360° videos.

**Figure 3:**
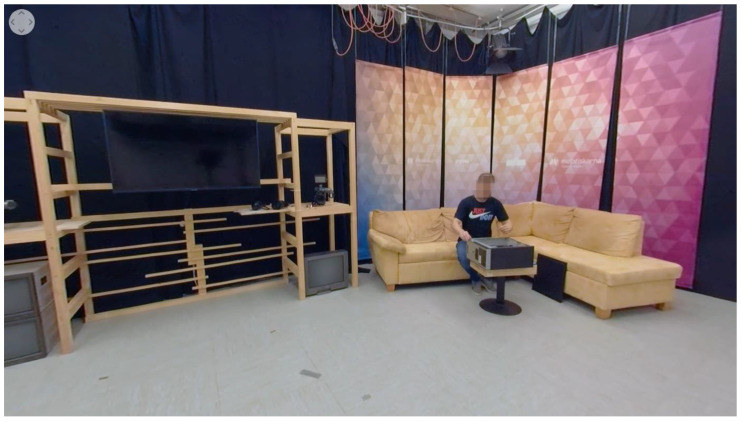
Screenshot of a chosen perspective from video 1 and video 2.

Video 1 represents the content for the control group. It shows a protagonist, sitting on the couch and repairing the computer (Figure 3). He opens it and starts repairing the computer hardware. The rest of the scene is static and does not provide any dedicated cues for guiding viewers’ attention toward the brand on the protagonist’s T-shirt. Neutral non-diegetic ambient music is added in the background. Video 2 (cue: sound) represents the same “mise-en-scene” as video 1 with a different auditory part, where the non-spatial sound of repairing the computer is included and serves as a diegetic cue for searching its source, thus guiding viewers’ attention towards the protagonist and the brand.

**Figure 4:**
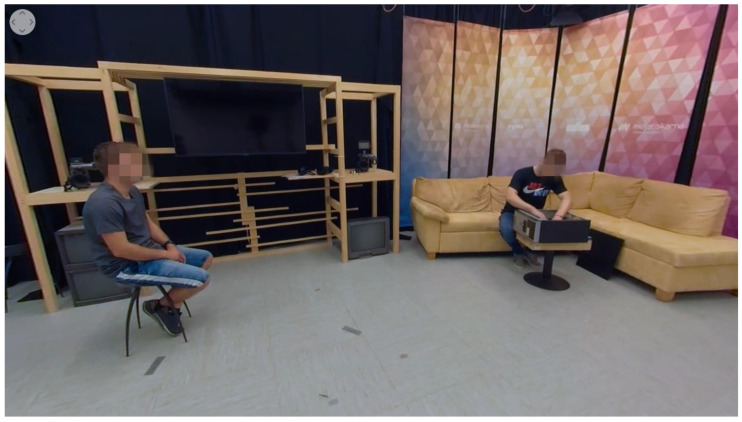
Screenshot of a chosen perspective from video 3.

Video 3 (method: head pointing) depicts the protagonist, who is sitting on the couch and repairing the computer, while another character is sitting on the other side of the studio and watching him working (Figure 4). His head is pointed toward the protagonist and the branded t-shirt, where head pointing is used as a diegetic guidance method, directed toward the brand. The rest of the scene does not include other dedicated diegetic guidance methods.

**Figure 5:**
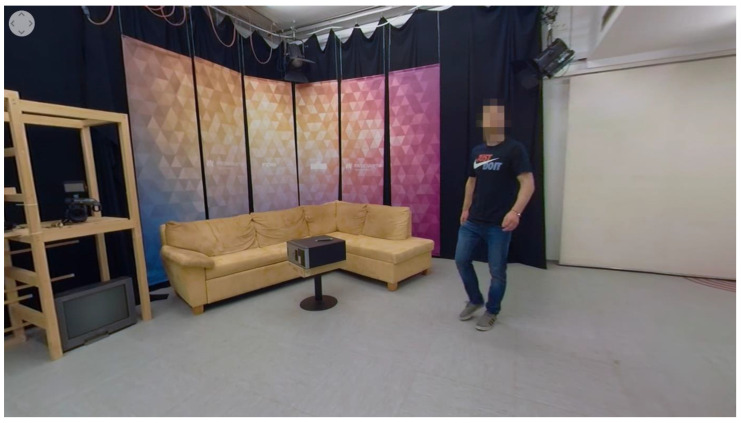
Screenshot of a chosen perspective from video 4 (cue: moving protagonist).

Video 4 (method: moving protagonist) shows the protagonist, who opens the studio door and goes past the camera to the other part of the scene (Figure 5). He grabs and adjusts the light settings, then adjusts the cameras while returning towards the exit. Despite the movement, the brand on the protagonist’s t-shirt is visible. The moving protagonist is used as a diegetic method for guiding viewers’ attention toward the brand. The rest of the scene does not include other dedicated diegetic guidance methods.

### 3.3. Ethics

Users of the SurveyCircle platform could access the questionnaire comprising the relevant instructions and variables. Initially, they were not informed about the specific goals of the study, as this may have influenced the outcomes of being focused consciously on the placed brands [20]. The study was designed according to the ethical Standards and Guidelines from The Declaration of Helsinki [57]. The Ethics Committee approval was unnecessary, as our study was performed in the form of an online survey, and volunteers/participants were ensured total anonymity. This is also in line with the related studies on product placement and 360° video [20,55,58]. The only personal data were age and gender, whereas there were no issues concerning endangerment of life, health, dignity, integrity, right to self-determination, privacy, or confidentiality of personal information of the research subjects. Before the data collection, participants had to provide permission for the data collection by agreeing with statements related to the purpose of the survey, their voluntary approach, and the anonymity of the results.

### 3.4. Measuring Instrument

A demographic part of the questionnaire asked participants about gender, age, and control variables—a device that will be used for watching the 360° video, and the experience of watching the 360° video. The latter was adapted from prior studies [20,59] and consisted of a 7-point differential scale ranging from (1) “no experience at all” to (7) “a lot of experience”.

The following section was programmed so that participants were assigned to one of four videos randomly (independent variables), and instructions were adapted to the device they had chosen for playing the video. In this light, they were informed to either click and drag (PC), move the device (smartphone/tablet), or move the head (HMD) to change the visual perspective.

The next page included the dependent variables of brand recall and brand recognition that were adapted from prior studies [20,60]. Returning to the previous page was disabled to prevent the possibility of recalling the brand by watching the video again. Participants were informed to answer the following questions just from their memory. Brand recall was obtained by asking participants which brand they can remember from the video. The correctly recalled brand was coded as 1, while wrong brands or missing fields were coded as 0. On the next page, participants were asked about brand recognition, where they had to choose the brand they had seen while watching the video. Among the right brands, there were two mock or dummy brands from the same category, and the option “others/do not remember”. The correctly recognized brand was coded as 1, while other options were coded as 0. In the end, another control variable was included—brand familiarity. It was adapted from prior studies [61,62] and consisted of a 7-point differential scale ranging from (1) “low familiarity” to (7) “high familiarity”.

## 4. Results

Three hundred and sixty respondents were assigned randomly to either video 1 (no cue), video 2 (sound), video 3 (head pointing), or video 4 (moving protagonist). The numbers of respondents per video were 88, 85, 95, and 93, respectively. Among the participants, 229 were females, 126 were males, and six identified as others. Participants were aged between 15 and 65 (average age = 27). The majority of respondents watched 360° video by means of a PC (*n* = 289), followed by Smartphone/Tablet (*n* = 69) and HMD (*n* = 3). The descriptive statistics of other ordinal control and dependent measures are reported in Table 1.

The Shapiro-Wilk test was used to test the distribution of the variables of brand recognition and brand recall, which was essential for choosing an appropriate test for statistical analysis. The test showed that the distributions of brand recall and brand recognition were significantly different from a normal distribution (*p* < 0.001). In turn, the non-parametric Chi-Square Test of Homogeneity was used, to determine whether there was a difference in product placement effectiveness between the three groups of guidance cues and the control group.

The lowest percentage of brand recall was in video 3 (head pointing), while video 4 (moving protagonist) resulted in the highest percentage of brand recall (Table 2). Despite the differences, the brand recall scores of the four independent groups were not statistically significantly different (df = 3; *p* = 0.887). Thus, **H1(a)** was not confirmed.

In terms of brand recognition, there were higher scores in all the groups. Video 1 (no cues) resulted in the lowest percentage of recognized brands, while video 4 (moving protagonist) again had the highest score (Table 2). However, the brand recognition scores of the four independent groups were not statistically significantly different (df = 3; *p* = 0.675), meaning that **H1(b)** was not supported.

The results mean that we have not proven the effectiveness of one diegetic guiding cue over the other according to brand recall and brand recognition. A binominal logistic regression was performed, to ascertain the effects of control variables—age, experience, gender, device, brand familiarity, and video type, on the likelihood that participants recall and recognize the brand. The important assumptions for conducting the test were met, namely, dichotomous dependent variables, more independent variables, independence of observations, more than 15 cases per independent variable, a linear relationship between the continuous (also ordinal) independent variables, and the logit transformation of the dependent variables, and no significant outliers.

In terms of brand recall, the logistic regression model was not statistically significant, χ2(10) = 13.949, *p* = 0.175. Out of six independent variables, only brand familiarity added significantly to the model/prediction (*p* = 0.04; Table 3). It means that increasing brand familiarity was associated with an increased likelihood of recalling the brand. For each unit of brand familiarity, the odds of recalling the brand increased by a factor of 1.247. The Cochran-Armitage test of the trend was used in addition, to assess the linear trend between brand familiarity and brand recall. The levels of familiarity were from 1-low familiarity to 7-high familiarity, and the proportion of respondents recalling the brand was 0%, 25%, 0%, 43%, 20%, 46%, and 41%, respectively (Table 4). The Cochran-Armitage test of trend showed a statistically significant linear trend, *p* = 0.04. Nevertheless, the results should be taken cautiously, as respondents` ratings were more concentrated toward higher brand familiarity ratings.

The same statistical procedure was applied for brand recognition, where the logistic regression model was not statistically significant, χ^2^(10) = 14.629, *p* = 0.146. None of the six predictor variables was statistically significant (Table 5).

## 5. Discussion

Prior studies on visual attention in 360° video technology outlined important insights into attention guidance methods’ effectiveness [8,9], while there is a lack of exploring its effects in the case of the unconscious nature of placed brand memory. This study explores the influence of different attention guidance methods on product placement effectiveness in 360° video. The participants were assigned randomly to one of the four videos, with three different diegetic guiding cues and one video for the control group.

Our results did not support the effectiveness of diegetic guidance methods for enhancing placed brand memory in 360° video, which is inconsistent with related studies that substantiate the methods’ favorable effects for directing viewers’ attention [8,25,52]. The reasons for the discrepancy with related studies are twofold. The first lies in the unconscious and unobtrusive nature of product placement, where the placed brands are of secondary importance to the viewer. Related studies on diegetic guidance considered guiding viewers’ attention in a certain direction, generally without focusing on unconscious memory [8,25], such as brand recall and recognition. The second reason may represent the technology condition, where the majority of respondents of the present study used a PC device to interact with 360° video, while related studies are focused on more immersive technology conditions (HMD) [26].

The results suggest that directing viewers’ attention by means of different diegetic guiding cues may direct viewers’ eyes to a certain direction; however, the cognitive focus and visual attention might not follow it [34], since it can depend on other factors, such as the audience involvement and technology condition. In addition, attention is limited by the processing speed of visual information and visual working memory [37,38]. Product placement usually works on an unconscious level [27], which made it more challenging for remembering the brand and measuring the brand memory, especially when considering that directing viewers’ attention may be influenced by their task or goal (top-down approach) [30].

Participants generally had higher recognition than recalling scores, indicating that brand memory was stimulated better when they had to recognize the brand among others from the same category rather than recall it freely. Although the results were not statistically significant, the guiding cue with a moving protagonist turned out to have the highest brand recall and recognition scores. This may be due to the constant visual tracking of a moving subject wearing a branded t-shirt, where the action may have stimulated more interest in the narrative and more prominent brand occurrence as the protagonist was closer to the camera. It associates with a related study suggesting the higher recall rates of prominent placements over the subtle placements in 360° video [12].

Our studies also considered certain control variables, where brand familiarity was found to influence brand recall positively. This finding is consistent with related product placement studies in video games [50,51], suggesting that respondents who were more familiar with the placed brand were more likely to recall it after watching the video.

### 5.1. Policy Implications

This study also raises the issue of the product placement policy that should follow more advanced media formats. On one hand, the Audiovisual Directive in Europe allows product placement practices [44], and different states define product placement regulations and disclosure [38,39,40]. On the other, disclosures are rarely considered seriously in practice [47], and there is even less policy attention to emerging technologies and media formats (e.g., video games, 360° videos). The latter may require new approaches to disclosing the sponsored content, due to the different product placement outcomes in emerging media contexts [14]. In the context of the present study, we suggest improving the policy by providing directions for disclosing sponsored content in emerging media formats (e.g., 360° video) and defining the appropriateness of disclosure and guiding cues` incorporation. For instance, while logos for disclosing the commercial message should be non-diegetic and exposed overtly at a certain part of the program, methods for guiding viewers` attention towards the brand should be limited to subtle and diegetic cues that are in line with the unobtrusiveness of the product placement.

### 5.2. Limitations and Future Directions

Although our study has not confirmed the differences in the effectiveness of certain diegetic guiding methods for product placement effectiveness in 360° video, the results and limitations provide important directions for future research.

The participants watched one of the 90-s long 360° videos. This length may not be sufficient to evaluate the effectiveness of certain guiding methods, as guidance of attention was found to be challenging at the beginning of new scenes [9], and the brand exposure duration can also influence the outcomes [27]. Accordingly, viewers may not have been involved with the content and the brand enough to be guided unconsciously by the diegetic guiding cues, considering that our videos included a 90-s-long shot of one scene. Thus, we suggest using longer videos for future research, where the level of involvement with the content and brand derived from the in-game advertising [63] should be adapted to 360° video and considered in this context.

Since brand familiarity showed a positive correlation with the recalled brand in our study, future research on product placement in 360° video should explore further the associations between brand familiarity and recalling rates for a higher number of brands. Other control variables did not influence the outcomes. However, our study did not focus on equally distributed participants across the control variables, so the influences should be examined further in future studies. For instance, although brand memory did not differ according to the device for playing the video, participants were not distributed equally across the technology condition, where only three watched it via HMD. Future research should, thus, consider the potential effectiveness of diegetic guiding cues in more immersive technology conditions, as they are substantiated to be more effective when using the HMD [8]. Diegetic guidance should also be considered for prominent and subtle brands separately, as a related study shows different recalling scores according to brand prominence [12].

This study was also limited to specific diegetic guiding cues, whereas other examples could return different outcomes (e.g., moving lights, a new source of sound, and small protagonists’ gestures). On the other hand, more overt or non-diegetic guiding cues could likely result in higher brand memory, but exploring the latter would interfere with the concept of product placement concerning the unobtrusive brand placement [17] that should influence the viewers’ on an unconscious level without disturbing the story [16].

The questionnaire with the incorporated videos provided a sufficient sample size (*n* = 360) for comparing four different categorical groups. However, the interpretation of the results derives from the assumption that all the participants watched the entire length of the 360° video. We suggest an experimental design with enforced internal validity and control for future studies, where fewer guiding methods should be applied due to possible smaller sample sizes. In addition, brand memory is not connected directly with visual attention. An eye-tracking methodology should be applied to explore the deviations between visual attention and brand memory [39,64].

## 6. Conclusions

The paper exposes the coherent perspectives between diegetic guiding methods, 360° video, and product placement. Diegetic guidance is a suitable method for directing viewers’ attention in 360° video, and is substantiated as appropriate for the unobtrusive nature of product placement. However, our findings suggest that the chosen methods are ineffective guidance in the context of unconscious brand memory, namely, brand recall and brand recognition. The present study thus exposes the discrepancy with the results of related studies on viewers’ attention, by emphasizing that directing viewers` attention by subtle and diegetic methods is not necessarily effective for the unconscious memory of information that is of secondary importance to the viewer (product placement). It also stresses the importance of brand familiarity and provides important policy implications and future research directions regarding the guiding methods in relation to the technology condition, brand familiarity, and video type.

## Figures and Tables

**Table 1:** Descriptive statistics of control and dependent variables.

	Minimum	Maximum	Mean	Std. Deviation
Experience	1	7	3.07	1.686
Brand familiarity	1	7	6.39	1.171
Brand recall	0	1	0.389	0.488
Brand recognition	0	1	0.496	0.5

**Table 2:** The share of recalled and recognized brands.

		Video 1	Video 2	Video 3	Video 4	Total
Brand recall	No	53 (60.2%)	52 (61.2%)	61 (64.2%)	54 (58.7%)	220 (61.1%)
	Yes	35 (39.8%)	33 (38.8%)	34 (35.8%)	38 (41.3%)	140 (38.9%)
	Sum	88 (100%)	85 (100%)	95 (100%)	92 (100%)	360 (100%)
Brand recognition	No	47 (53.4%)	43 (50.6%)	50 (52.6%)	42 (45.2%)	182 (50.4%)
	Yes	41 (46.6%)	42 (49.4%)	45 (47.4%)	51 (54.8%)	179 (49.6%)
	Sum	88 (100%)	85 (100%)	95 (100%)	93 (100%)	361 (100%)

**Table 3:** Logistic Regression Predicting Likelihood of Brand Recall.

	B	SE	Wald	df	*p*	Odds Ratio	95% CI for Odds Ratio	
							Lower	Upper
Age	−0.016	0.013	1.498	1	0.221	0.984	0.959	1.010
Gender			0.308	2	0.857			
Gender 1	20.600	15,867.039	0.000	1	0.999	884,391,009.353	0.000	.
Gender 2	20.471	15,867.039	0.000	1	0.999	776,950,463.953	0.000	.
Experience	−0.010	0.067	0.021	1	0.885	0.990	0.869	1.129
Device			1.496	2	0.473			
Device 1	−1.472	1.292	1.297	1	0.255	0.230	0.018	2.890
Device 2	−1.589	1.314	1.462	1	0.227	0.204	0.016	2.682
**Familiarity**	**0.221**	**0.109**	**4.060**	**1**	**0.044**	**1.247**	**1.006**	**1.545**
Video			0.640	3	0.887			
Video 1	0.023	0.313	0.005	1	0.942	1.023	0.554	1.888
Video 2	−0.020	0.314	0.004	1	0.950	0.980	0.530	1.814
Video 3	−0.198	0.306	0.418	1	0.518	0.820	0.450	1.495

**Table 4:** The proportion of respondents recalling the brand according to brand familiarity.

	Level of Brand Familiarity							
		1	2	3	4	5	6	7
Brand recall	No	5 (100%)	3 (75%)	2 (100%)	9 (56%)	23(79%)	31 (54%)	147 (60%)
	Yes	0 (0%)	1 (25%)	0 (0%)	7 (44%)	6 (21%)	26 (46%)	100 (40%)

**Table 5:** Logistic Regression Predicting the Likelihood of Brand Recognition.

	B	SE	Wald	df	*p*	Odds Ratio	95% CI for Odds Ratio	
							Lower	Upper
Age	−0.020	0.012	2.653	1	0.103	0.980	0.956	1.004
Gender			0.904	2	0.636			
Gender 1	21.115	16,009.269	0.000	1	0.999	1480219689.658	0.000	
Gender 2	20.899	16,009.269	0.000	1	0.999	1192237494.774	0.000	
Experience	0.007	0.065	0.011	1	0.918	1.007	0.886	1.144
Device			0.438	2	0.804			
Device 1	−0.808	1.257	0.413	1	0.520	0.446	0.038	5.237
Device 2	−0.757	1.277	0.351	1	0.553	0.469	0.038	5.728
Familiarity	0.108	0.098	1.232	1	0.267	1.114	0.920	1.349
Video			1.198	3	0.754			
Video 1	−0.293	0.307	0.908	1	0.341	0.746	0.408	1.363
Video 2	−0.176	0.307	0.328	1	0.567	0.839	0.459	1.531
Video 3	−0.279	0.298	0.881	1	0.348	0.756	0.422	1.355

## Data Availability

The data from all the subjects are freely available on DOI: 10.17632/73d8v3ztsj.1.
